# Identification of a Potential Regulatory Variant for Colorectal Cancer Risk Mapping to Chromosome 5q31.1: A Post-GWAS Study

**DOI:** 10.1371/journal.pone.0138478

**Published:** 2015-09-18

**Authors:** Juntao Ke, Jiao Lou, Xueqin Chen, Jiaoyuan Li, Cheng Liu, Yajie Gong, Yang Yang, Ying Zhu, Yi Zhang, Jing Gong

**Affiliations:** State Key Laboratory of Environment Health (Incubation), MOE (Ministry of Education) Key Laboratory of Environment & Health, Ministry of Environmental Protection Key Laboratory of Environment and Health (Wuhan), and Department of Epidemiology and Biostatistics, School of Public Health, Tongji Medical College, Huazhong University of Science and Technology, Wuhan, China; Medical College of Soochow University, CHINA

## Abstract

Large-scale genome-wide association studies (GWAS) have established chromosome 5q31.1 as a susceptibility locus for colorectal cancer (CRC), which was still lack of causal genetic variants. We searched potentially regulatory single nucleotide polymorphisms (SNPs) in the overlap region between linkage disequilibrium (LD) block of 5q31.1 and regulatory elements predicted by histone modifications, then tested their association with CRC via a case-control study. Among three candidate common variants, we found rs17716310 conferred significantly (heterozygous model: OR = 1.273, 95% confidence interval (95%CI) = 1.016–1.595, *P* = 0.036) and marginally (dominant model: OR = 1.238, 95%CI = 1.000–1.532, *P* = 0.050) increase risk for CRC in a Chinese population including 695 cases and 709 controls. This variation was suggested to be regulatory altering the activity of enhancer that control *PITX1* expression. Using epigenetic information such as chromatin immunoprecipitation-sequencing (ChIP-seq) data might help researchers to interpret the results of GWAS and locate causal variants for diseases in post-GWAS era.

## Introduction

In China, colorectal cancer (CRC) is the fifth most commonly diagnosed cancer in males and the third in females, with an estimated 310,244 new cases and 149,722 deaths occurring in 2011 [1]. Risk factors for CRC include diet, physical inactivity, obesity, smoking and drinking [2, 3], and it’s well established that genetic factors also play an important role in the etiology of CRC [4, 5]. By now, genome-wide association studies (GWAS) and fine mapping researches have identified risk variants mapping to over 30 independent susceptibility loci of CRC in Europeans and Asians [6–20]. However, vast majority of these variants reside in intergenic and intronic regions, and the most likely biological mechanism that links them to disease is regulatory [21].

Accumulating evidence showed that non-coding genetic variants of risk-associated loci could exert an effect on gene expression by modulating the activity of regulatory elements [22], including promoters, enhancers, insulators and silencers. And various histones in the flanking nucleosomes of such genomic regions have been discovered to carry characteristic post-translational modifications [23, 24]. For example, promoters are usually marked by H3K4me3 (histone H3 trimethylated at lysine 4) and enhancers by H3K4me1 (histone H3 monomethylated at lysine 4), and either is additionally marked by H3K27ac (histone H3 acetylated at lysine 27) upon activation [25–28]. Today, genome-wide mapping of histone modifications accomplished by chromatin immunoprecipitation-sequencing (ChIP—seq) is widely used to predict promoters and enhancers [29–32].

5q31.1 was first mapped as a CRC susceptibility locus by Jia et al [17] in both East Asian and European populations, further supported by another larger-scale genetic study by Zhang et al [20] in East Asians. The most likely involved gene *PITX1* (paired-like homeodomain 1) has been considered to be a tumor suppressor gene relating to carcinogenesis of CRC [33, 34] and other cancers [33, 35–38]. However, the reported strongest risk polymorphism rs647161 is of unclear function and not in any known transcribed or regulatory sequences. So, we reasoned that rs647161 is not the causal single nucleotide polymorphism (SNP) and the real functional SNPs remain to be mined in this region. At the same time, identifying functional SNPs that overlap tissue-specific regulatory elements predicted by chromatin status such as histone modifications, have represented a powerful approach to progress from statistical association to functionality and causality in post-GWAS genetic researches [39–42].

In this study, we analyzed ChIP-seq data of histone modifications from Encode project [29], explored potentially regulatory variants within the susceptibility locus 5q31.1, and investigated candidate common SNPs’ association with CRC risk via a case-control study in Chinese population.

## Material and Methods

### Study Participants

A total of 695 CRC cases and 709 cancer-free controls were recruited from Tongji Hospital of Huazhong University of Science and Technology (HUST) between 2008 and 2011. All subjects were unrelated ethnic Han Chinese living in Wuhan City and its surrounding areas. The inclusion criteria for cases were histopathologically confirmed CRC without previous chemotherapy or radiotherapy, and no restriction to gender and age. Controls were selected randomly from a physical examination programs at the same hospital in the same time period as the patients were enrolled, part of which were also involved in our pervious studies [43, 44], and were adequately matched to cases in terms of gender and age (±5 years). Herein, smokers were defined as those who had smoked at least one cigarette per day for 12 months or longer at any time of their life, while non-smokers were defined as those who had not. At recruitment, 5-ml peripheral venous blood was collected from each subject after a written informed consent was obtained. This study was approved by ethnics committee of Tongji Hospital of Huazhong University of Science and Technology.

### Selection of Candidate SNPs

Candidate SNPs in this study are identified as common (minor allele frequency, MAF>0.05) genetic variants locating in the overlap region between the 5q31.1 locus and CRC-specific regulatory elements marked by proper epigenetic marks. Firstly, we downloaded the genotype information of Han Chinese in Beijing, China (CHB) that was 500kb upstream and downstream of the tagSNP rs647161 from HapMap database, and input that data into the software HaploView to obtain the linkage disequilibrium (LD) block of rs647161 with the criteria of *r* ^*2*^>0.8, which was defined as the boundary of GWAS locus 5q31.1. Secondly, we acquired ChIP-seq data of different histone modifications produced in two CRC cell lines HCT116 and Caco2 from UCSC database integrating with Encode data (S1 Table), then extracted the extent of their signal peaks standing for regulatory elements, where we intersected two replication versions of the same data set (intersection) and united all different data sets (union). Thirdly, basing on dbSNP database, we picked out the SNPs with MAF>0.05 in CHB that lie in the overlapping region between aforementioned LD block and peaks. Finally, three SNPs, rs2193941, rs17716310 and rs7703385 were chosen as candidate SNPs for the next-step genotyping.

### Genotyping

Genomic DNA was extracted from peripheral blood leukocytes using RelaxGene Blood System DP319-02 (Tiangen, Beijing, China) by reference to the manufacturer’s instructions. All SNPs were genotyped with the TaqMan SNP Genotyping Assay (Applied Biosystems, Foster City, CA, USA) on a 7900HT Fast Real-Time PCR System (Applied Biosystems, Foster City, CA, USA). 5% duplicated samples were randomly selected to assess the reproducibility for quality control, with a concordance rate of 100%.

### Statistical Analysis

The t test and χ^2^ test was applied to estimate differences in variables and distributions of genotypes between cases and controls. Hardy-Weinberg equilibrium (HWE) was evaluated by applying the goodness-of-fit χ^2^ test in controls. The strength of association between each SNP and CRC risk was measured by the odds ratio (OR) and its corresponding 95% confidence interval (95%CI). In order to avoid the assumption of genetic models, heterozygous, homozygous, dominant, recessive and additive models were analyzed. The statistical test power of each SNP was calculated by POWER v3.0 (http://www.mds.qmw.ac.uk/stat-gen/dcurtis/software.html). And ORs and corresponding 95%CIs, adjusted by gender, age and smoking status were calculated by unconditional multivariate logistic regression. Statistical analyses were performed using SPSS Software v20.0 (SPSS, Chicago, Illinois, USA). The potential gene-environment and SNP-SNP interactions were evaluated by a pair-wise analysis under multiplicative [45] and additive interaction models [46]. The *P* values for multiplicative interaction were calculated using a multiplicative interaction term under the multivariate logistic regression model in SPSS software. And the *P* values for additive interaction were assessed by a bootstrapping test of goodness-of-fit using Stata v11.0 (Stata Corporation, College Station, TX). All *P* values were two sided with the statistical significance criteria of *P* < 0.05.

## Results

### Selection of Candidate SNPs

The area of GWAS susceptibility loci 5q31.1 we defined by LD was chromosome 5: 134467220–134518445. After a three-step bioinformatics analysis, three common polymorphisms, rs2193941, rs17716310 and rs7703385 that situated within the peaks of histone modification ChIP-Seq data generated from HCT116 or Caco2, were found in the above loci (Table 1).

**Table 1:** Candidate regulatory SNPs in GWAS locus 5q31.1.

SNP	Position (hg19)	Major/Minor Allele	CHB MAF	Overlapping Peaks	Histone Modification	Cell Line
rs2193941	134469594	A/G	0.28	134469520–134469630	H3k4me3	Caco2
rs17716310	134476759	A/C	0.31	134475122–134478405	H3k4me1	HCT116
				134475349–134477528	H3k27ac	HCT116
rs7703385	134478074	C/G	0.28	134475122–134478405	H3k4me1	HCT116

Abbreviations: CHB, Han Chinese in Beijing, China; MAF, minor allele frequency.

### Population Characteristics

695 incident cases and 709 frequency-matched controls were enrolled in this study. As shown in Table 2, the proportion of males was 58.42% in cases compared with 56.42% in controls (*P* = 0.449, Pearson χ^2^ = 0.570). Mean age and corresponding standard deviation was 60.16±12.26 years for cases and 59.80±13.18 years for controls (*P* = 0.598 by t test), and there was no statistically significant differences between cases and controls in terms of age distribution (*P* = 0.305, Pearson χ^2^ = 3.625) among four categories (≤50, 51–60, 61–70 and ≥71). As expected, more smokers were presented in the cases than in the controls (35.25% versus 29.62%; *P* = 0.022, Pearson χ^2^ = 5.257), considering that cigarette smoking was a well-established risk factor for CRC (2).

**Table 2:** The characteristics of the study population.

	Cases	Controls		
	No. (%)	No. (%)	χ2	P
Total	695	709		
Gender			0.570	0.449
Male	406 (58.42)	400 (56.42)		
Female	289 (41.58)	309 (43.58)		
Age (mean±SD)	60.16±12.26	59.80±13.18		0.598^a^
Agegroup			3.625	0.305
≦50	150 (21.58)	154 (21.72)		
51–60	207 (29,78)	181 (25.53)		
61–70	184 (26.47)	209 (29.48)		
≧71	154 (22.16)	165 (23.27)		
Smoking Status				
Non-Smoker	448(64.65)	499(70.38)	5.257	0.022
Smoker	245(35.35)	210(29.62)		

Abbreviations: SD, standard deviation.

^a^ *P* value was calculated by the *t* test.

### Association Analysis

All three SNPs, rs2193941, rs17716310 and rs7703385, were in HWE (*P*_HWE_ = 0.55, 0.17 and 0.55), and the statistical test power was 95.9%, 95.3% and 95.5% respectively. The genotype distributions of investigated polymorphisms were shown in Table 3. In association analysis, only rs17716310 showed significant association with CRC under heterozygote model, while the other two SNPs rs2193941 and rs7703385 presented no statistical evidence of relation to CRC risk.

**Table 3:** Association between individual SNP and colorectal cancer risk.

Genotype	Controls (%)	Cases (%)	*P* ^a^	OR (95%CI)^b^	*P* ^b^
***rs2193941***					
AA	310(44.4)	277(41.3)	0.516	1.000	
AG	305(43.7)	310(46.3)		1.142 (0.909–1.434)	0.253
GG	83(11.9)	83(12.4)		1.102 (0.779–1.560)	0.583
Dominant model				1.131(0.912–1.403)	0.263
Recessive model				1.040(0.750–1.442)	0.813
Additive model				1.078(0.921–1.263)	0.350
***rs17716310***					
AA	337(48.4)	294(43.1)	0.117	1.000	
AC	284(40.7)	314(46.0)		**1.273 (1.016–1.595)**	**0.036**
CC	76(10.9)	74(10.9)		1.104 (0.771–1.581)	0.589
Dominant model				**1.238(1.000–1.532)**	**0.050**
Recessive model				0.987(0.702–1.388)	0.939
Additive model				1.123(0.958–1.318)	0.153
***rs7703385***					
CC	324(47.2)	294(43.2)	0.310	1.000	
CG	290(42.3)	313(45.9)		1.194 (0.952–1.497)	0.125
GG	72(10.5)	74(10.9)		1.117 (0.776–1.606)	0.553
Dominant model				1.176(0.949–1.458)	0.138
Recessive model				1.031(0.730–1.457)	0.862
Additive model				1.103(0.939–1.296)	0.232

Abbreviations: OR, Odds ratio; 95%CI, 95% confidence interval.

^a^ P values were calculated by the Pearson Chi-Square test

^b^ Data were calculated by logistic regression model after adjusting for sex, age group and smoking status.

The nominal significant and marginal results were in bold.

Under multivariate logistic regression model adjusted for gender and age, individuals with AC genotype of rs17716310 had a significantly increased risk of CRC (OR = 1.273, 95%CI = 1.016–1.595, *P* = 0.036) compared to those with AA homozygote. A dominant model was performed to improve statistical power by combining the AC with CC into a C-carrier group (AC plus CC), and it showed that the allele C carriers got a marginal effect on CRC susceptibility (OR = 1.238, 95%CI = 1.000–1.532, *P* = 0.050). However, no significant risk of the variant C allele was seen in homozygous, recessive or additive model. As for rs2193941 and rs7703385, there were no positive results under all genetic models we studied.

### Analysis of Linkage Disequilibrium

**Fig 1:**
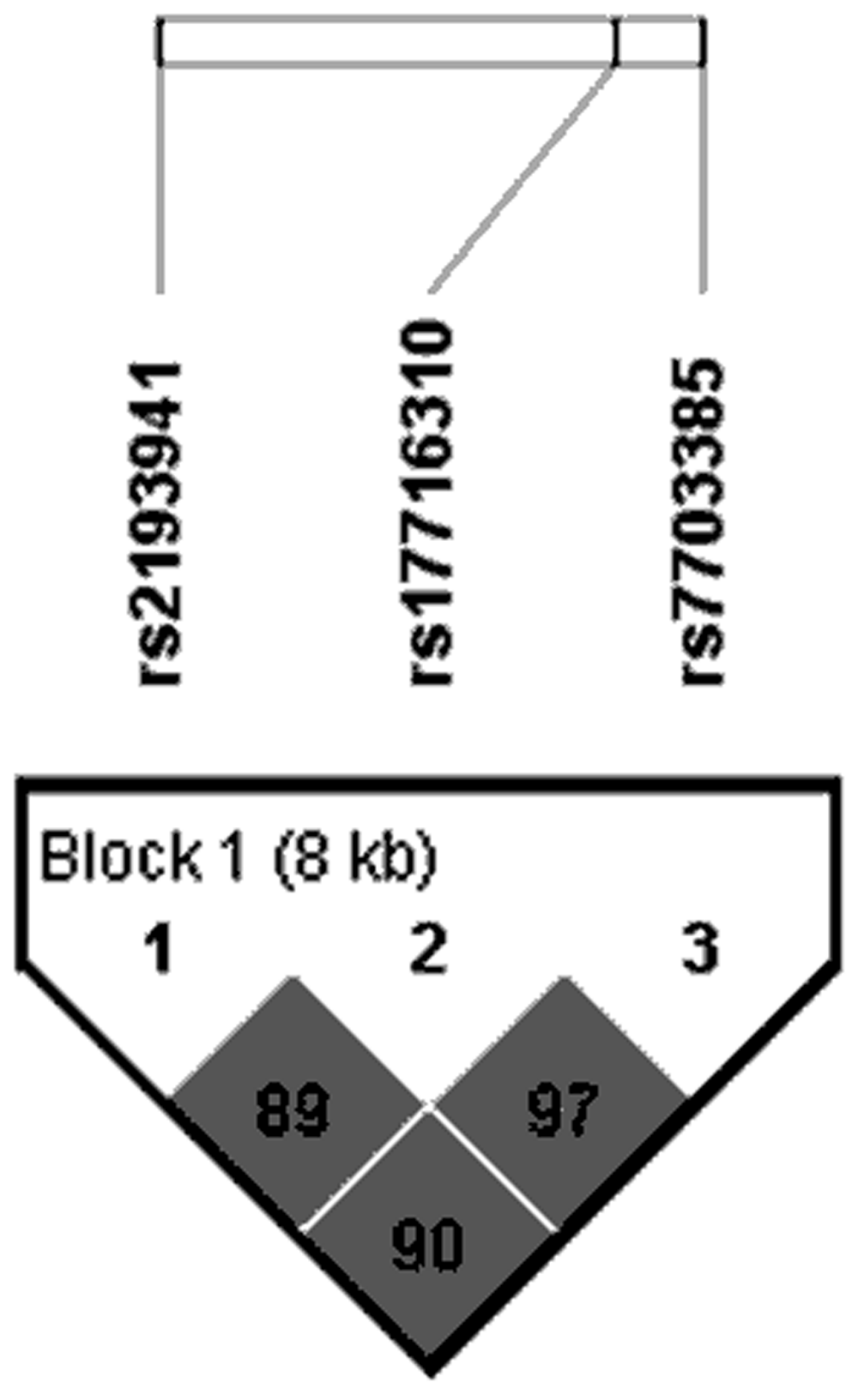
The LD block constructed by rs2193941, rs17716310 and rs7703385.

Shown in Fig 1, three investigated SNPs were in high LD with each other (rs2193941 and rs17716310, *r* ^*2*^ = 0.89; rs17716310 and rs7703385, *r* ^*2*^ = 0.97; rs2193941 and rs7703385, *r* ^*2*^ = 0.90) in our study. On the other hand, rs2193941, rs17716310 and rs7703385 were discovered to be in high LD with the tagSNP rs647161 (*r* ^*2*^ = 0.83, *r* ^*2*^ = 0.88, *r* ^*2*^ = 0.88, respectively) in CHB population of 1000 Genomes Project Phase 3.

### Interaction Analysis

Table 4 detailed the results of interaction analysis between the promising SNP rs17716310 and smoking, where we observed a significant interaction (*P*_mult_ = 0.013) in multiplicative terms. When we examined the pair-wise interactions among three candidate variations, we found no positive outcomes under both multiplicative and additive model (S2 Table).

**Table 4:** Interaction analysis between smoking and rs17716310 associated with CRC risk.

Smoking stusus	Genotype	Case/Control	OR (95%CI)^a^	*P*_mult_ ^a^	*P*_add_
Non-smoker	AA	189/232	1.000	**0.013**	0.238
	AC+CC	250/257	1.192 (0.919–1.547)		
Smoker	AA	104/105	1.281 (0.886–1.853)		
	AC+CC	137/103	1.712 (1.196–2.451)		

*P*_mult_ was calculated using the multiplicative interaction term.

*P*_add_ was calculated using the additive interaction model.

^a^ Data were calculated by logistic regression model after adjusting for gender and age group.

The nominal significant results were in bold.

## Discussion

In post-GWAS era, identifying specific functional genetic variants that actually accounts for phenotype is the major purpose and challenge, and regulatory elements of genome can help. Using epigenetic marks obtained from relevant cell types, we searched potentially functional SNPs that were situated in putatively regulatory elements, and validated their association with CRC in an independent population.

In this study, through combining GWAS locus 5q31.1 and promising regulatory regions predicted by histone modifications in CRC cell lines, we screened out three common variants, rs2193941, rs17716310 and rs7703385, in their overlap. After we conducted an association study in a Chinese population containing 695 CRC cases and 709 health controls, we found significant and marginal effect of rs17716310, which was in LD with tagSNP rs647161 and might interacted with smoking.

The findings led us to assume rs17716310 influenced CRC risk by altering the activity of regulatory elements that control *PITX1* expression. Lying within a region of the genome exhibiting chromatin modifications H3k4me1 and H3k27ac, rs17716310 is highly suggested to be a regulatory variants belonging to an active enhancer [47, 48]. It is approximately 107 kb upstream of the closest gene *PITX1*, which has been reported as a tumor suppressor downregulating the RAS pathway [33], activating *TP53* [49] and tuning telomerase activity [50]. In addition, lower *PITX1* expression has been found in human cancer tissue samples and cell lines [35–37], and associated with poor survival in CRC patients [51]. On the other side, in a online database HaploReg [52], rs17716310 was indicated to change the binding motif of p300 that functions as a transcriptional coactivator and histone acetyltransferase regulating gene expression by remodeling chromatin [53]. The variant might alter the binding site of some transcription factor(s), and impact on interaction between this active enhancer and the downstream promoter of *PITX1*, therefore impair transcription and expression of the suppressive gene, and consequently facilitate CRC tumorigenicity and susceptibility. As for the interaction with smoking found in multiplicative model, it might be due to the relations between smoke status and RAS pathway, *TP53* and telomerase activity [54–57] in which *PITX1* was involved. However, the assumption needs further functional experiments to be verified.

The application of epigenetic biofeature information such as histone modification ChIP-seq data to identify candidate enhancers have represented a useful tool to identify candidate functional SNPs in regulatory regions [58, 59], and databases such as UCSC and Encode have provided easy access to massive amounts of relevant data. Integrating newly arisen epigentics and traditional molecular epidemiology could be an effective approach to help interpreting GWAS resluts and discover causal variants for diseases in post-GWAS studies. Applying similar strategy to other CRC GWAS regions should assist in deeper understanding of CRC risk.

Still, several limitations should be acknowledged here. First, the strategy of retrieving candidate polymorphisms depended on the prediction from ChIP-seq data of two CRC cell lines, which was not rigorous enough to define exact regulatory elements, and not comprehensive enough to discover all functional SNPs inside. Second, the sample size of our case-control study was relatively small. Third, insufficient environmental and clinical information restricted us to further investigate the interactions between gene and other factors. Forth, lacking of functional experiments, biological reality beneath the statistically significant association we reported is uncertain.

In summary, we discovered a probably regulatory SNP in high LD with the GWAS tagSNP that is associated with CRC risk in Chinese population. Systematic researches on more susceptibility loci with greater sample sizes and follow-up functional analyses are warranted to identify causal variants and elaborate the biological mechanism of genetic etiology.

## Supporting Information

S1 Table ChIP-seq datasets downloaded from UCSC integrating Encode data. (DOC)

S2 Table Interaction analysis between rs17716310 and rs2193941, rs17716310 and rs7703385, rs2193941 and rs7703385. (DOCX)
