# Supplementary material for: Identification of a Potential Regulatory Variant for Colorectal Cancer Risk Mapping to Chromosome 5q31.1: A Post-GWAS Study
Source: PLoS One. 2015 Sep 18;10(9):e0138478. doi: 10.1371/journal.pone.0138478 (PMC4575091; doi:10.1371/journal.pone.0138478)
Supplement: S1 Table — (DOC) [file pone.0138478.s001.doc]

S1 Table. ChIP-seq datasets downloaded from UCSC integrating Encode data.

| Cell line | Histone Modification | Dataset |
| --- | --- | --- |
| Hct116 | H3k4me1 | wgEncodeSydhHistoneHct116H3k04me1UcdPk.narrowPeak |
| Hct116 | H3k27ac | wgEncodeSydhHistoneHct116H3k27acUcdPk.narrowPeak |
| Hct116 | H3k4me3 | wgEncodeUwHistoneHct116H3k4me3StdPkRep1.narrowPeak |
|  |  | wgEncodeUwHistoneHct116H3k4me3StdPkRep2.narrowPeak |
| Caco2 | H3k27me3 | wgEncodeUwHistoneCaco2H3k27me3StdPkRep1.narrowPeak |
|  |  | wgEncodeUwHistoneCaco2H3k27me3StdPkRep2.narrowPeak |
| Caco2 | H3k36me3 | wgEncodeUwHistoneCaco2H3k36me3StdPkRep1.narrowPeak |
|  |  | wgEncodeUwHistoneCaco2H3k36me3StdPkRep2.narrowPeak |
| Caco2 | H3k4me3 | wgEncodeUwHistoneCaco2H3k4me3StdPkRep1.narrowPeak |
|  |  | wgEncodeUwHistoneCaco2H3k4me3StdPkRep2.narrowPeak |
