# Supplementary material for: Identification of a Potential Regulatory Variant for Colorectal Cancer Risk Mapping to Chromosome 5q31.1: A Post-GWAS Study
Source: PLoS One. 2015 Sep 18;10(9):e0138478. doi: 10.1371/journal.pone.0138478 (PMC4575091; doi:10.1371/journal.pone.0138478)
Supplement: S2 Table — (DOCX) [file pone.0138478.s002.docx]

S2 Table. Interaction analysis between rs17716310 and rs2193941, rs17716310 and rs7703385, rs2193941 and rs7703385.

| Genotype | Genotype | Case/Control | OR (95%CI)^a^ | *P*_mult_^a^ | *P*_add_ |
| --- | --- | --- | --- | --- | --- |
| rs17716310 | rs2193941 |  |  |  |  |
| AA | AA | 266/307 | 1.000 | 0.199 | 0.472 |
| AA | AG+GG | 20/20 | 1.160 (0.609-2.211) |  |  |
| AC+CC | AA | 4/0 | -- |  |  |
| AC+CC | AG+GG | 367/359 | 1.180 (0.946-1.472) |  |  |
| rs17716310 | rs7703385 |  |  | 0.104 | 0.582 |
| AA | CC | 285/318 | 1.000 |  |  |
| AA | CG+GG | 4/4 | 1.085 (0.266-4.419) |  |  |
| AC+CC | CC | 2/1 | 1.989 (0.177-22.345) |  |  |
| AC+CC | CG+GG | 378/351 | 1.202 (0.967-1.493) |  |  |
| rs2193941 | rs7703385 |  |  | 0.372 | 0.487 |
| AA | CC | 268/297 | 1.000 |  |  |
| AA | CG+GG | 4/0 | -- |  |  |
| AG+GG | CC | 18/18 | 1.099 (0.559-2.162) |  |  |
| AG+GG | CG+GG | 367/361 | 1.127 (0.903-1.406) |  |  |

*P*_mult_ was calculated using the multiplicative interaction term.

*P*_add_ was calculated using the additive interaction model.

^a^ Data were calculated by logistic regression model after adjusting for gender and age group.
